# Albendazole and Corticosteroids for the Treatment of Solitary Cysticercus Granuloma: A Network Meta-analysis

**DOI:** 10.1371/journal.pntd.0004418

**Published:** 2016-02-05

**Authors:** Bing-Cheng Zhao, Hong-Ye Jiang, Wei-Ying Ma, Da-Di Jin, Hao-Miao Li, Hai Lu, Hideaki Nakajima, Tong-Yi Huang, Kai-Yu Sun, Shu-Ling Chen, Ke-Bing Chen

**Affiliations:** 1 Department of Clinical Medicine, Zhongshan School of Medicine, Sun Yat-sen University, Guangzhou, China; 2 Department of Orthopedics, the Third Affiliated Hospital of Southern Medical University, Guangzhou, China; 3 Department of Parasitology, Zhongshan School of Medicine, Sun Yat-sen University, Guangzhou, China; 4 Key Laboratory for Tropical Disease Control, Sun Yat-sen University, Guangzhou, China; 5 Department of Anesthesiology, the Sun Yat-sen Memorial Hospital of Sun Yat-sen University, Guangzhou, China; 6 Department of Orthopedics and Rehabilitation Medicine, Fukui University of Medical Sciences, Fukui, Japan; Universidad Peruana Cayetano Heredia, PERU

## Abstract

**Background:**

Solitary cysticercus granuloma (SCG) is the commonest form of neurocysticercosis in the Indian subcontinent and in travelers. Several different treatment options exist for SCG. We conducted a Bayesian network meta-analysis of randomized clinical trials (RCTs) to identify the best treatment option to prevent seizure recurrence and promote lesion resolution for patients with SCG.

**Methods and Principal Findings:**

PubMed, EMBASE and the Cochrane Library databases (up to June 1, 2015) were searched for RCTs that compared any anthelmintics or corticosteroids, alone or in combination, with placebo or head to head and reported on seizure recurrence and lesion resolution in patients with SCG. A total of 14 RCTs (1277 patients) were included in the quantitative analysis focusing on four different treatment options. A Bayesian network model computing odds ratios (OR) with 95% credible intervals (CrI) and probability of being best (*P*_best_) was used to compare all interventions simultaneously. Albendazole and corticosteroids combination therapy was the only regimen that significantly decreased the risk of seizure recurrence compared with conservative treatment (OR 0.32, 95% CrI 0.10–0.93, *P*_best_ 73.3%). Albendazole and corticosteroids alone or in combination were all efficacious in hastening granuloma resolution, but the combined therapy remained the best option based on probability analysis (OR 3.05, 95% CrI 1.24–7.95, *P*_best_ 53.9%). The superiority of the combination therapy changed little in RCTs with different follow-up durations and in sensitivity analyses. The limitations of this study include high risk of bias and short follow-up duration in most studies.

**Conclusions:**

Dual therapy of albendazole and corticosteroids was the most efficacious regimen that could prevent seizure recurrence and promote lesion resolution in a follow-up period of around one year. It should be recommended for the management of SCG until more high-quality evidence is available.

## Author Summary

Neurocysticercosis is an infection of the central nervous system by the larva of *Taenia solium* (pork tapeworm). It is a leading cause of epilepsy in the world. The disease takes many different forms, each with different optimal treatment. In this study, we focused on the treatment of solitary cysticercus granuloma (SCG), previous evidence on which is inconclusive. Since many different regimens have been compared in clinical trials of SCG, we conducted a network meta-analysis. This method is powerful as it can analyze quantitatively all of the data from all comparisons together. The result can tell us how different treatments perform against each other and how treatments should be ranked. The outcomes of our meta-analysis suggest that the combination of albendazole and corticosteroids is the most efficacious regimen to control seizures in affected patients and to promote the total disappearance of the lesion, compared with albendazole alone, corticosteroids alone, and conservative treatment.

## Introduction

Neurocysticercosis (NCC), a parasitic disease of the nervous system caused by *Taenia solium* (pork tapeworm), is a leading cause of acquired epilepsy worldwide [1, 2]. The disease is widely prevalent around the world, and has pleomorphic clinical and radiologic manifestations [1]. Solitary cysticercus granuloma (SCG), presenting as a single small enhancing lesion, is found in ~20% of NCC cases in endemic areas, and is the commonest type of NCC in the Indian subcontinent as well as in travelers of industrialized countries returning from endemic zones [3, 4]. SCG has traditionally been considered the degenerating form of long-established vesicular cyst that cannot maintain immune evasion and thus is under the host’s immune attack. A recent hypothesis proposes that SCG represents fresh infection that is rapidly detected and destroyed by the host’s immune system. [5]

Treatment might be different for patients with live and degenerative/dead parasite. While there is sufficient information in support of the use of the combination of anthelmintics and corticosteroids in patients with viable cystic parenchymal NCC [6–10], the treatment of SCG has not been optimally defined [11]. Besides, the recent American Academy of Neurology (AAN) evidence-based guideline on NCC didn’t address management issues of different types of lesion independently [12]. Currently, the overall evidence from randomized clinical trials (RCTs) on drug therapy for SCG consists of comparisons between the combination of anthelmintics and corticosteroids therapy, anthelmintics therapy alone, corticosteroids therapy alone and conservative treatment (limited to treatment of symptoms), such as antiepileptic drugs alone without anthelmintics or corticosteroids. Several pairwise meta-analyses have evaluated the independent efficacies of anthelmintics and of corticosteroids [9, 13, 14]. However, multiple different regimens have never been compared with each other simultaneously.

The network of evidence can be better examined in a mixed treatment comparison framework with Bayesian method [15, 16]. This approach fully respects randomization, accounts for the correlation of multiple observations within the same trial, and allows estimation of relative efficacies of different drugs and their combination. Here, we systematically reviewed and analyzed RCTs on drug therapy for SCG and conducted a Bayesian network meta-analysis to determine the effect of different therapies on seizure control and on radiological resolution of the disease.

## Methods

The protocol of this study was determined according to the Cochrane Collaboration and PRISMA statement [17].

### Search strategy

We searched the electronic databases of PubMed, EMBASE and the Cochrane Library (from inception until June 1, 2015) without restrictions on language or publication date. The logic combinations of the following terms were searched in the Title/Abstract: *cysticercosis*, *neurocysticercosis*, *solitary cysticercus granuloma*, *single small enhancing computed tomographic lesion*, *cysticidal*, *anticysticercal*, *anthelmintic*, *albendazole*, *praziquantel*, *corticosteroid*, *steroid*, *prednisolone*, *methylprednisolone*, and *dexamethasone*. The computer retrieval was supplemented by manual search of reference lists of identified studies and (systematic) reviews on neurocysticercosis.

### Study selection

The identified citations were initially screened at the title and abstract level, and then retrieved as full-text copies if they reported potentially relevant studies. To be included in the analysis, studies had to be randomized clinical trials (RCTs) that included patients with new onset seizures and diagnosed with SCG based on clinical and imaging studies according to the accepted criteria [18]. All studies compared the efficacy of anthelmintics (albendazole and/or praziquantel) or corticosteroids, or both, head to head or with placebo or no drugs. Studies were excluded if they compared different dosages or durations of the same medication, if only patients with cystic or multiple enhancing lesions were included, and if none of the quantitative outcomes of interest (*see below*) were reported. Studies using concomitant drugs, such as antiepileptic drugs (AEDs) were not excluded if balanced among the trial arms. When more than one report describing the same study were published, the one with the most recent or complete data was used for meta-analysis.

### Data collection

Two researchers independently reviewed the studies with disagreements in eligibility, methodological quality or data extraction resolved through discussion and consensus. Data were collected for each eligible RCT on study characteristics, patient characteristics, and outcome results. The goal of this study was to compare the efficacies of different drug therapies in the following aspects: seizure recurrence, defined as the occurrence of one or more convulsions after the beginning of treatment, and lesion resolution, defined as complete disappearance of the granuloma with no residual scar, calcification or perilesional edema on imaging studies, by MRI or CT scan. If a study reported outcomes at multiple time points, only data from the most recent follow-up were extracted for analysis.

### Quality assessment

The methodological quality of the included RCTs was appraised using the Cochrane Collaboration’s tool for assessing risk of bias [19], which consists of seven items: sequence generation; allocation concealment; blinding of participants and personnel; blinding of outcome assessors; incomplete outcome data; selective outcome reporting; and other bias. Blinding and incomplete outcome data were assessed separately for the two primary outcomes. The overall risk of bias of a trial was expressed as low, moderate, or high.

### Statistical analysis

Therapeutic interventions were included in quantitative analyses if they had been studied in at least two trials. We conducted Bayesian network meta-analysis using the binomial likelihood model for multi-arm trials [20, 21], given the outcomes were dichotomous and included multi-arm trials. Our model adopted random effects because it is the most appropriate and conservative analysis to account for variance among trials. The Markov Chains Monte Carlo method was used for analysis. Three Markov chains ran simultaneously with different initial values. 150,000 simulations were generated for each of the three sets of initial values, with the first 50,000 discarded to avoid the influence of initial unstable values. The convergence was assessed with trace plots and the Brooks-Gelman-Rubin statistic.

The odd ratios (OR) were estimated from the median of the posterior distribution and the accompanying 95% credible intervals (CrI), which can be interpreted in the same manner as the conventional 95% confidence interval (CI). For comparison, the estimates from just the head-to-head evidence for each pair of comparison were also worked out with the Mantel-Haenszel method of the conventional pairwise meta-analysis.

Furthermore, for each outcome, we estimated the probability that each treatment regimen was the most, the second, the third, and the least efficacious, based on their ranks in each iteration of Markov chain. These probability values were used for generating cumulative probability plots and calculating the Surface Under the Cumulative RAnking curve (SUCRA), with 1 representing the best treatment and 0 the worst [22].

We examined the validity of the network models by evaluating three of their important characteristics. The goodness of model fit was measured by the posterior mean of the residual deviance, which should be close to the data points when the model can provide adequate fit. Heterogeneity was defined as the variability of the results across trials. It was estimated from the posterior median between-study variance τ^2^, with τ^2^ < 0.04 indicating a low level of heterogeneity and τ^2^ > 0.40 a high level [23]. Consistency, defined as agreement between direct and indirect sources of evidence, was first assessed visually by comparing the results of network meta-analysis and pairwise meta-analysis, and then tested statistically by calculating the ratio of two odds ratios (RoR) from direct and indirect evidence in each closed loop in the network of interventions. RoR values close to 1 mean that the two sources are in agreement [24].

The considerable variation in follow-up duration among the included RCTs and the fact that probability of both primary outcomes are related with time [25] did not allow calculation of the absolute rate difference and number needed to treat for each intervention by using the baseline rates across the conservative treatment arms. Considering that evidence may be different from RCTs with different follow-up duration, we performed meta-regression analysis with follow-up duration (≤ 6 months versus ≥ 9 months for seizure recurrence, 3 months versus 6 months for lesion resolution) as an interaction [26]. We calculated the subgroup interaction term β and checked whether its 95% credible interval included the possibility of no interaction. We performed further sensitivity analysis by sequentially removing one study at a time from the overall dataset. A *post hoc* analysis was performed to compare different treatments on the risk of residual calcification during the evolution of SCG lesions.

Assessment of publication bias using the funnel plots was precluded by the small number of studies included in the meta-analysis.

Conventional pairwise meta-analysis was performed with Review Manager 5.3.3 (Cochrane Collaboration, Nordic Cochrane Centre, Denmark). Network meta-analysis including meta-regression analysis was performed with winBUGS 1.4.3 (MRC Biostatistics Unit, Cambridge, UK). Test for consistency was conducted with Stata 12.0 (StataCorp LP, College Station, TX).

## Results

### Literature search

**Fig 1:**
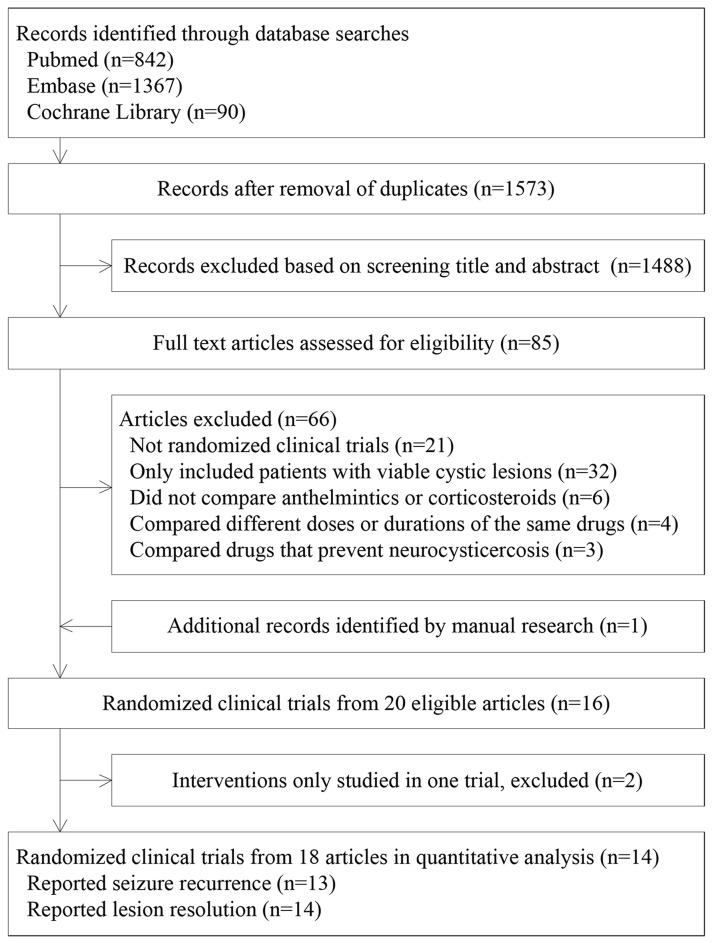
Flowchart of the selection process of the RCTs.

Fig 1 is a flow chart of the study and summarizes the process of trial selection. Twenty articles reporting 16 RCTs met the inclusion criteria [27–42]. The included RCTs covered six different treatment regimens for SCG: albendazole (evaluated in 5 trials), praziquantel (1 trial), corticosteroids (9 trials), albendazole plus corticosteroids (6 trials), albendazole and praziquantel plus corticosteroids (1 trial), and conservative treatment (11 trials). The two praziquantel-containing regimens were evaluated only in one trial, so that they and the corresponding trials were not suitable for the network meta-analysis.

### Characteristics of the randomized clinical trials

The main features of the RCTs included in the quantitative analysis are summarized in Table 1. Fourteen trials involving 1,277 randomized patients were included. All the included RCTs were conducted in India where cysticercosis is highly endemic. The proportion of women ranged from 29.7% to 47.2%, and the mean age of patients at baseline ranged from 7.4 to 24 years. Each participant in each RCT, but two, was diagnosed with a solitary enhancing lesion. In one RCT [31], only 70.8% of the patients had a single enhancing lesion while the others carried two or more lesions. However, it was possible to extract the data of patients with single lesions, thus allowing the inclusion in the analysis of data of only these patients. In another trial [32], some patients (18%) had two rather than one enhancing lesions and the outcome data could not be separated. We decided to include this trial given the small proportion of patients with two lesions. All trials had two arms, except one in which the three active treatments were compared directly with each other [40]. The dosage of albendazole and corticosteroids were similar across the trials, but the duration of treatment varied from 3 to 28 days. Antiepileptic drugs were used in all trials. The follow-up period ranged from 6 to 18 months for seizure recurrence and from 2 to 6 months for lesion resolution.

**Table 1:** Main characteristics of the randomized clinical trials included in the quantitative analysis.

Author, year	Patients (n = 1277)		Interventions^a^			Follow-up	Outcome		Risk of bias^b^
	n (M, F)	Age, yrs	Anthelmintics	Corticosteroids	Concomitant treatment		Seizure recurrence	Complete resolution	
Padma et al., 1994	75 (52, 23)	21.8	1. Albendazole: 1 wk	None	AED	CT scans after 1 and 3 m	NA	8/40	NA/
			2. Placebo	None	AED		NA	8/35	Moderate
Baranwal et al., 1998	72 (34, 29)	7.4	1.Albendazole: 4 wk	Pred 1–2 for 5d	AED	CT scans after 1 and 3 m, 15 m TFU at 3 m intervals	7/31	20/31	Low/Low
			2. Placebo	Pred 1–2 for 5d	AED		11/32	12/32	
Gogia et al., 2003	72 (38, 34)	1.5–12	1.Albendazole: 4 wk	Pred 2 for 3d	AED	CT scan after 6 m, 6 m TFU	3/24	11/18	Low/Low
			2. Placebo	Pred 2 for 3d	AED		5/27	9/18	
Kalra et al., 2003	123 (65, 58)	7.6	1.Albendazole: 4 wk	Dexamethasone 0.15 mg/kg/d for 5d	AED	CT scan after 3 m, 6 m TFU at 3 m interval	6/45	14/45	High/High
			2. None	None	AED		15/45	9/45	
Mall et al., 2003	108 (56, 41)	22	1. None	Pred 1 for 10 d, tapered off in next 4 d	AED	CT scans after 1 and 6 m, 6 m TFU at 1 m intervals	1/49	43/49	High/High
			2. None	None	AED		6/48	25/48	
Singhi et al., 2004	133 (66, 44)	1–14	1.Albendazole: 4 wk	None	AED	CT scans after 3 and 6 m, 18 m TFU at 3 m intervals	5/37	28/37	High/High
			2.Albendazole: 4 wk	Pred 2 for 1 wk	AED		4/35	26/35	
			3. None	Pred 2 for 3 wk tapered off in wk 4	AED		14/38	29/38	
Garg et al., 2006	60 (39, 21)	13.5	1. None	Pred 1 for 10 d, tapered off in next 4 d	AED	CT scan after 6 m, 9 m TFU at 1 m intervals	4/30	16/30	Low/Low
			2. None	Placebo	AED		14/30	14/30	
Prakash et al., 2006	52 (36, 16)	16	1. None	IV methylpred 1 g/1.72 m^2^/d for 5d	AED	CT scan after 2 m, 9 m TFU at 1 m intervals	4/25	15/25	High/High
			2. None	None	AED		9/27	5/27	
Kishore et al., 2007	100	NR	1. None	Pred 1 for 10 d	AED	CT scan after 2–3 m, 12 m TFU	5/47	32/47	High/High
			2. None	Placebo	AED		12/45	24/45	
Sharma et al., 2007	90 (52, 38)	19.3	1. Albendazole: 15 d	Pred 1 for 2 wk tapered off in next 3 d	AED	CT scans after 1 and 6 m, 6 m TFU at 1 m intervals	9/48	33/45	High/High
			2. None	Pred 1 for 2 wk tapered off in next 3 d	AED		5/42	25/36	
Thussu et al., 2008	54 (28, 15)	24	1. Albendazole: 2 wk	None	AED	CT scans after 1, 3 and 6 m, 6 m TFU	3/23	22/23	High/High
			2. None	None	AED		4/20	14/20	
De Souza et al., 2009	123 (59, 44)	19.6	1. Albendazole: 4 wk	None	AED	MRI after 3, 6 and 12 m, 12 m TFU	7/50	10/45	High/High
			2. None	None	AED		5/53	9/48	
Chaurasia et al., 2010	67 (43, 24)	17	1. Albendazole: 3 d	None	AED	CT scan after 6 m, 6 m TFU	3/33	28/33	High/High
			2. Placebo	None	AED		1/34	14/34	
Singla et al., 2011	148 (104, 44)	19	1. None	Pred 40–60 mg/d for 2 wk, tapered off in next 4 d	AED	CT scan after 3 m, MRI after 6 m, 9 m TFU at 3 m intervals	16/73	28/60	Low/High
			2. None	Placebo	AED		19/75	21/54	

^a^ In all studies, the dose of albendazole was 15 mg/kg body weight/day. The dose of prednisolone is in mg/kg/day unless otherwise indicated. All patients were receiving AED monotherapy (phenytoin or carbamazepine).

^b^ The first assessment is for the outcome seizure recurrence, and the second one is for lesion resolution.

AED = antiepileptic drug, TFU: total follow-up, NA = not applicable, NR = not reported, wk: week, d; day, M: males, F: females, Pred: prednisolone.

There was high risk of selection bias in most studies because they used random number tables to generate random number sequences with no or unclear method of allocation concealment. The performance bias was high in more than half of the studies due to lack of blinding of participants. Blinding of seizure assessment was unclear or non-existent in those studies too, but for the assessment of lesion resolution, blinding was generally well maintained. S1 Fig shows the assessment process of the risk of bias of the studies included in this meta-analysis.

### Seizure recurrence

**Fig 2:**
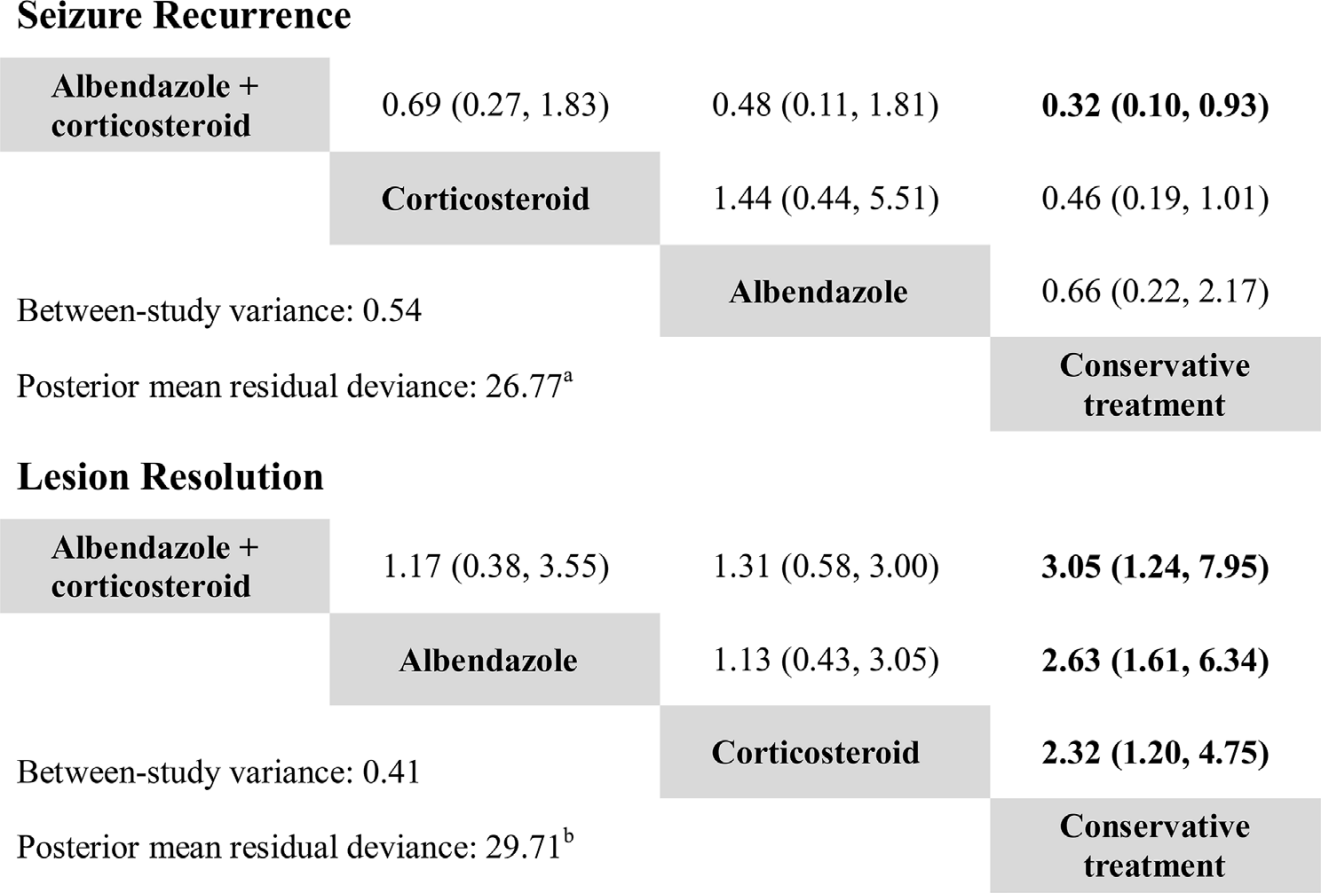
Pooled odds ratios for seizure recurrence and lesion resolution in Bayesian network meta-analysis. In each cell, odd ratios (with 95% credible intervals) are the pooled effects of the intervention labeled horizontally to the left of the plot compared with the intervention labeled vertically below. Results with statistical significance are shown in bold type. ^a^ Compared with 27 data points. ^b^ Compared with 29 data points.

**Fig 3:**
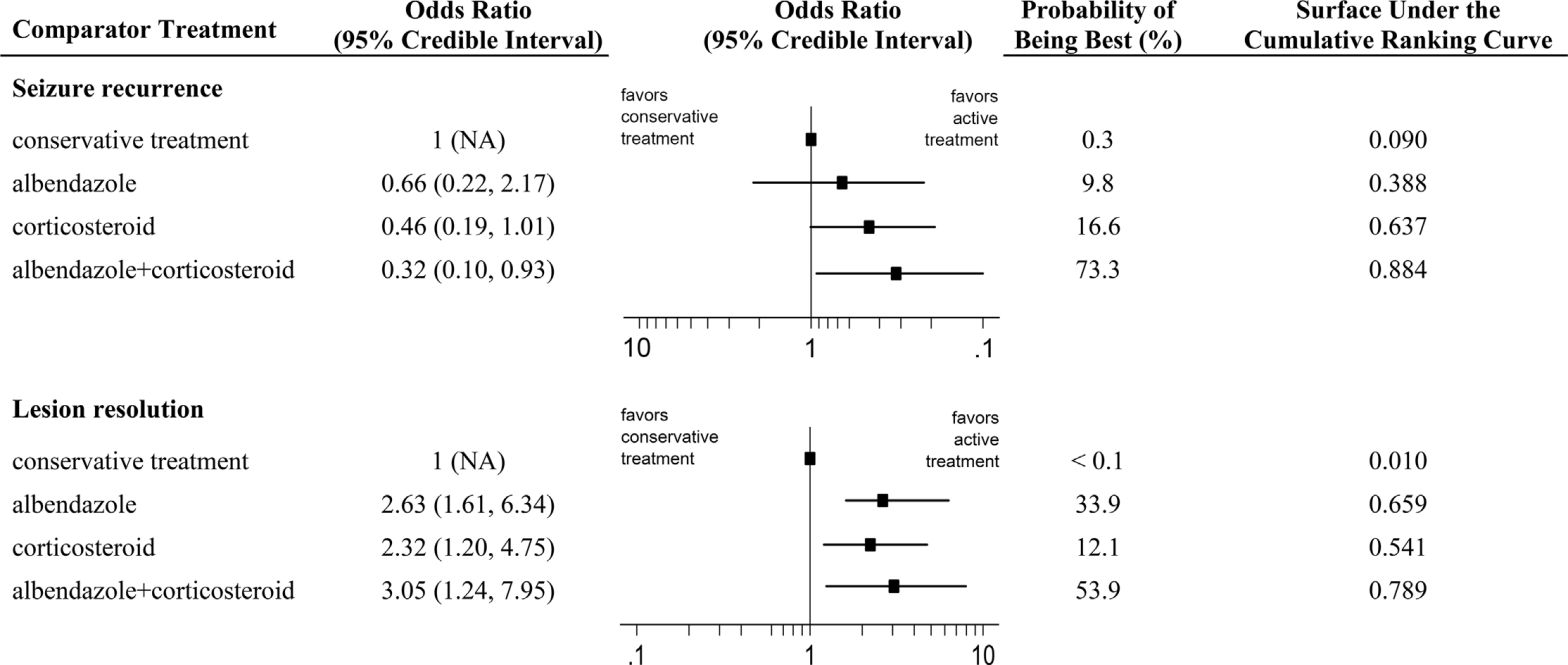
Results of the Bayesian network meta-analysis for seizure recurrence and lesion resolution. Pooled odds ratios with 95% credible intervals, probability of being the best treatment, and the surface under the cumulative ranking curve were presented. NA = not applicable.

Thirteen RCTs were used for the quantitative analysis of seizure recurrence. The network diagram for this outcome is presented in S2(A) Fig. Network meta-analysis showed that albendazole plus corticosteroids was the only treatment protocol that significantly decreased the recurrence of seizure during the follow-up period compared with conservative treatment (OR 0.32, 95% CrI 0.10–0.93, Figs 2 and 3). The results were similar in the only direct comparison RCT that evaluated albendazole plus corticosteroid versus conservative treatment (OR 0.31, 95% CI 0.11–0.89) [32]. The risk reduction for corticosteroids alone was marginal outside the level of significance (0.46, 0.19–1.01), and the efficacy of albendazole alone did not even approach statistical significance (0.66, 0.22–2.17). While there were no significant differences among the three active treatments, the ranking probabilities and cumulative probability plots indicated that the combination of albendazole and corticosteroids was superior to either treatment alone (Fig 3). The combination therapy had the greatest probability of being the best treatment (*P*_best_ 73.3%), and the SUCRA values were 0.884, 0.637, and 0.388 for albendazole plus corticosteroid, corticosteroid, and albendazole, respectively. A test of subgroup interaction between RCTs with follow-up period of ≥9 months and those with ≤ 6 months was not statistically significant (subgroup interaction term β 0.05, -1.73–1.77), adding support to the conclusion that the combination of the two groups of RCTs was not inappropriate.

The fit of model was good with the posterior mean of the residual deviance of 26.77, compared with 27 data points. However, the estimated between-study variance was 0.54 (0.03–2.52), which is potentially considerable with its uncertainty caused by the relatively small number of studies. Visual inspection of the results from pairwise and network meta analyses showed obvious inconsistency between direct and indirect estimates for the contrast albendazole versus corticosteroids, and this was confirmed by a large RoR value (3.15) of the corresponding loop in the network (S3(A) Fig). Since only one RCT [40] supplied direct evidence for the comparison, we investigated the inconsistency by removing this trial in a sensitivity analysis. The result is presented in S1 Table. The combination of albendazole plus corticosteroid (0.31, 0.11–0.76) and corticosteroid alone (0.37, 0.17–0.68) both significantly reduced the risk of seizure recurrence, with combination therapy being the better one in probability analysis (*P*_best_ 67.1% versus 31.8%, SUCRA 0.882 versus 0.767). The probability analysis suggested that the efficacy of albendazole monotherapy was even worse than conservative treatment although there was no significant difference between the two (OR conservative treatment versus albendazole 0.73, 0.23–2.23). Note that the pooled estimates of network meta-analysis generally overlapped with the results of conventional pairwise meta-analysis (when available) and that the estimated between-study variance decreased from 0.54 to 0.16.

### Lesion resolution

The outcome of lesion resolution was analyzed in all 14 RCTs. The network diagram is presented in S2(B) Fig. In the network meta-analysis, compared with conservative treatment, the efficacy of albendazole plus corticosteroids combination therapy in inducing resolution of SCG was the highest (3.05, 1.24–7.95), followed by albendazole alone (2.63, 1.61–6.34), and corticosteroids alone (2.32, 1.20–4.75, Figs 2 and 3). The same order was also identified in conventional pairwise meta-analysis but the confidence intervals were wider and only the efficacy of corticosteroid therapy reached statistical significance. The differences between the three treatments were not conclusive. Nevertheless, the combination of albendazole plus corticosteroid (*P*_best_ 53.9%, SUCRA 0.789) was more likely the best treatment for this outcome in probability analysis, compared with the monotherapy of albendazole (33.9%, 0.659) and corticosteroid (12.1%, 0.541) (Fig 3).

The posterior mean residual deviance was close to the number of data points (29.71 compared with 29) and thus the model fit was adequate. Heterogeneity was high (between-study variance 0.41) but acceptable. RoR values all close to 1 demonstrated no significant inconsistency between direct and indirect evidence for any of the pairwise treatment comparisons (S3(B) Fig).

The effect of interaction between the trials with 3-month follow-up and those with 6-month was insignificant (subgroup interaction term β 0.40, -0.84 to 1.75), although the point estimate was positive, suggesting that the efficacy of the combination therapy in promoting lesion resolution could be more obvious in short follow-up. This is reasonable since many SCGs resolve spontaneously with time [25]. Sensitivity analyses by sequentially removing one study at a time yielded largely the same results.

### *Post hoc* analysis- residual calcification

During review of the literature, 8 studies were identified that included data describing the frequency of residual calcification on follow-up imaging [27–29, 31, 34, 35, 41, 42]. Since calcific residue is one of the major predictors for future seizure recurrence [25], we did an additional *post hoc* analysis to evaluate the effect of different therapies on reducing the risk of residual calcification of SCG. Only pairwise meta-analysis was conducted because a closed loop for network meta-analysis was not formed. All pooled ORs are close to 1 with wide 95% CIs (S4 Fig), indicating that none of regimens showed a better effect on reducing the risk of residual calcification compared with others.

## Discussion

SCG is the commonest form of NCC seen in India and high-income countries, and is also found in about 20% of NCC cases elsewhere [3–5]. Although the granuloma shows spontaneous resolution with time, complete resolution can take anywhere from a few weeks to several years [43]. Since 1993 when albendazole was first shown to hasten the resolution of long persistent SCG [44], several clinical trials have been conducted to evaluate the effects of albendazole and other treatment options. Based on a Bayesian network of 14 RCTs that included 1277 patients, the results of the present meta-analysis showed that the combination therapy of albendazole and corticosteroids for SCG reduced the risk of seizure recurrence by two thirds and tripled the odds of lesion resolution during a short follow-up period of around one year, compared with conservative treatment. Although the differences in the beneficial effects of the combination therapy of albendazole plus corticosteroids compared with either treatment alone did not reach statistical significance, the combined therapy consistently showed higher probabilities of being at superior ranking positions for both outcomes. The albendazole and corticosteroids monotherapies showed similar and significant efficacy in promoting lesion resolution, but their benefits failed to translate into better seizure outcome during the follow-up. The superiority of the combination therapy was robust and changed little in trials with different follow-up durations and in sensitivity analyses.

Previous meta-analyses [9, 10, 13] reported that anthelmintic therapy with albendazole improved the seizure-free rate and hastened the resolution of the granuloma. However, these analyses combined clinical trials with different comparison groups (anthelmintics versus conservative treatment, anthelmintics versus corticosteroids, combination of anthelmintics and corticosteroids versus conservative treatment, and combination of anthelmintics and corticosteroids versus corticosteroids) making it impossible to determine the efficacy of anthelmintics itself and of the combination therapy. In fact, only three studies directly compared albendazole alone with placebo or no drugs, and the pooled estimates showed borderline significant improvement in lesion resolution and no difference in seizure outcome. Our network meta-analysis confirmed that albendazole alone did not improve the seizure-free rate although more lesions showed radiological resolution. The efficacy of corticosteroids alone in the treatment of SCG was evaluated in two pairwise meta-analyses with inconsistent results [13, 14]. Both studies used the same set of trials, yet Otte et al. [13] in their meta-analysis incorrectly extracted the data from the trial by Kishore et al. [34]. They misused the data of the placebo arm for the prednisolone arm and that of prednisolone for the placebo, thus yielding pooled effects that fell just short of statistical significance. In our study, we also used the pairwise meta-analysis, which confirmed the benefits of corticosteroids monotherapy for both outcomes. These benefits remained significant in network meta-analysis, although the corticosteroids monotherapy tended to be inferior to the combination therapy and albendazole monotherapy in promoting lesion resolution and inferior to the combination therapy in preventing seizure recurrence. Based on RCTs and pairwise meta-analysis, an expert consensus on diagnostic and therapeutic schemes for SCG recommended a short course (1–2 weeks) of albendazole with or without corticosteroids be prescribed soon after the first seizure [11]. Our study suggests that albendazole alone may not be effective on seizure control, and that the combination therapy of albendazole and corticosteroids should be initiated to bring the most benefit for patients with SCG.

The observed effects of albendazole and corticosteroids are supported by their mechanisms of actions and the histopathology of the granulomatous lesion. The cysticercus granuloma consists of a dying parasite surrounded by fibrosis, angiogenesis and infiltration of inflammatory cells [45]. The parasite or its parts are still present, offering a target for the anthelmintics to act on. The attack on parasite accelerates its destruction and leads to a faster and more efficient lesion resolution, but at the same time hastens the release of parasitic antigens and exacerbates local inflammation [46]. The study by Robinson et al. demonstrated that substance P produced within cysticercosis granulomas is capable of inducing seizure activity [47]. The anti-inflammatory and immunosuppressive properties of corticosteroids seem to reduce the generation of the seizure-inducing mediators, limit the inflammatory damage to neural tissue and control perilesional edema. Corticosteroids also interact with albendazole by reducing the elimination rate of albendazole sulfoxide, the active component of albendazole, thus increasing its plasma concentrations [48]. The clinical synergism between albendazole and corticosteroids results in better seizure control as well as early resolution of the granulomatous lesion. However, because the analyzed clinical trials do not provide information on the timing of seizure recurrence in relation to drug administration, it is not clear whether the favorable seizure outcome achieved by the combination therapy is the result of a reduced likelihood of seizure activity during and shortly after the administration of albendazole and corticosteroids or due to a more sustained effect.

Our study has several limitations. First, the majority of RCTs included in the analysis were at high risk of bias mainly because of inadequate allocation concealment and blinding. Only four studies were considered to have low-to-moderate risk of bias for the two outcomes, respectively, so that sensitivity analyses with only high quality studies were not possible. Second, all included RCTs were conducted in India. It is not certain whether the conclusions of this study apply to other populations. Third, under each class of treatment, there were variations in dosage and duration of the drugs used. This might have introduced some heterogeneity into the network meta-analysis. Treating them as different regimens, however, would not be feasible due to the insufficient number of studies to form a well connected network. The optimal match of dosages and durations of albendazole and corticosteroids needs further research. Fourth, the duration of the follow-up period varied among the included RCTs, making it another source of heterogeneity. Previous meta-analyses have tried some resolutions to the problem, such as performing separate meta-analyses at different time points of follow-up [13] or extracting data in the form of number of events per person-years observed [49]. In fact, estimating person-year of follow-up in these trials is very imprecise, and to our knowledge, there are currently no suitable methods that allow inclusion of all time points in a network meta-analysis. Here we explored the effects of differences in the duration of the follow-up period by meta-regression. The follow-up period was found not to significantly influence the results. Nevertheless, the average duration of the follow-up period of the included RCTs was generally short. Currently we cannot make firm conclusions on the effects of therapies more than one year after treatment. Future studies should focus on the efficacy of treatment in long-term seizure recurrence and granuloma resolution. Finally, limited data were available for two praziquantel-containing regimens to include in the analysis. In one trial [38], 26 patients were assigned to receive single-day praziquantel therapy or no therapy. Complete resolution was found in 78% (11 out of 14) and 50% (6 out of 12) of patients, respectively. Another trial compared the combination of albendazole, praziquantel and prednisolone with the combination therapy of albendazole and prednisolone [33]. After 6-month follow-up, complete lesion resolution was observed in 72% (38 out of 53) of patients of the praziquantel-treated group, versus 52% (26 out of 50) of the control group. The differences were not statistically significant in both studies. Although a previous meta-analysis showed that praziquantel might be less effective than albendazole in the treatment of NCC [49], the two anthelmintics have different mechanisms of action and have synergistic effects when used in combination [50]. More data are required before praziquantel can be added to the combination of albendazole and corticosteroids therapy for the treatment of SCG.

Despite the above limitations, based on the comprehensive review and robust statistical method, our network meta-analysis provides a complete picture for the efficacy of different management options for patients with SCG. The combination of albendazole and corticosteroids performs better than other therapies in reducing seizure recurrence and promote lesion resolution during a follow-up period of around one year. Until more direct active comparisons are available, it should be recommended for the treatment of SCG.

## Supporting Information

S1 Checklist PRISMA Checklist. (DOC)

S1 Table Pooled odds ratios for seizure recurrence in sensitivity analysis after removal of the clinical trial by Singhi at el. (PDF)

S1 Fig Risk of bias assessment. (TIF)

S2 Fig Network diagrams of the comparisons in the network meta-analysis for seizure recurrence (A) and lesion resolution (B). The size of the nodes is proportionate to the number of patients (in parentheses) randomized to the treatment. The width of the lines is proportionate to the number of direct comparisons (beside the lines) between the connected treatments.
(TIF)

S3 Fig Inconsistency plot of the network meta-analysis for seizure recurrence (A) and lesion resolution (B). In a total of 8 loops of the two networks, none showed statistically significant inconsistency since all confidence intervals for RoRs were compatible with zero inconsistency (RoR = 1). However, for the loop “ABZ–CT–CS” in the network of seizure recurrence, the mean RoR is larger than 3, meaning that the direct estimate can be three times as large as the indirect estimate or the opposite. ABZ: albendazole, CS: corticosteroids, CT: conservative treatment, RoR: ratio of odds ratios.
(TIF)

S4 Fig Forest plots of pairwise meta-analyses on residual calcification. ABZ: albendazole, CS: corticosteroids, CT: conservative treatment.
(TIF)

## References

[1] Garcia HH, Nash TE, Del Brutto OH. Clinical symptoms, diagnosis, and treatment of neurocysticercosis. Lancet Neurol. 2014;13(12):1202–15. 10.1016/S1474-4422(14)70094-8 25453460 PMC6108081

[2] Singh G, Burneo JG, Sander JW. From seizures to epilepsy and its substrates: neurocysticercosis. Epilepsia. 2013;54(5):783–92. 10.1111/epi.12159 23621876

[3] Coyle CM, Mahanty S, Zunt JR, Wallin MT, Cantey PT, White AC Jr., et al Neurocysticercosis: neglected but not forgotten. PLoS Negl Trop Dis. 2012;6(5):e1500 10.1371/journal.pntd.0001500 22666505 PMC3362619

[4] DeGiorgio CM, Sorvillo F, Escueta SP. Neurocysticercosis in the United States: review of an important emerging infection. Neurology. 2005;64(8):1486; author reply. 15851761 10.1212/wnl.64.8.1486

[5] Garcia HH, Gonzalez AE, Rodriguez S, Tsang VC, Pretell EJ, Gonzales I, et al Neurocysticercosis: unraveling the nature of the single cysticercal granuloma. Neurology. 2010;75(7):654–8. 10.1212/WNL.0b013e3181ed9eae 20713953 PMC2931772

[6] Garcia HH, Pretell EJ, Gilman RH, Martinez SM, Moulton LH, Del Brutto OH, et al A trial of antiparasitic treatment to reduce the rate of seizures due to cerebral cysticercosis. N Engl J Med. 2004;350(3):249–58. 14724304 10.1056/NEJMoa031294

[7] Carpio A, Kelvin EA, Bagiella E, Leslie D, Leon P, Andrews H, et al Effects of albendazole treatment on neurocysticercosis: a randomised controlled trial. J Neurol Neurosurg Psychiatry. 2008;79(9):1050–5. 10.1136/jnnp.2008.144899 18495737

[8] Garcia HH, Gonzales I, Lescano AG, Bustos JA, Zimic M, Escalante D, et al Efficacy of combined antiparasitic therapy with praziquantel and albendazole for neurocysticercosis: a double-blind, randomised controlled trial. Lancet Infect Dis. 2014;14(8):687–95. 10.1016/S1473-3099(14)70779-0 24999157 PMC4157934

[9] Del Brutto OH, Roos KL, Coffey CS, Garcia HH. Meta-analysis: Cysticidal drugs for neurocysticercosis: albendazole and praziquantel. Ann Intern Med. 2006;145(1):43–51. 16818928 10.7326/0003-4819-145-1-200607040-00009

[10] Abba K, Ramaratnam S, Ranganathan LN. Anthelmintics for people with neurocysticercosis. Cochrane Database Syst Rev. 2010;(3):CD000215. 10.1002/14651858.CD000215.pub4 20238309 PMC6532590

[11] Singh G, Rajshekhar V, Murthy JM, Prabhakar S, Modi M, Khandelwal N, et al A diagnostic and therapeutic scheme for a solitary cysticercus granuloma. Neurology. 2010;75(24):2236–45. 10.1212/WNL.0b013e31820202dc 21172847 PMC3013586

[12] Baird RA, Wiebe S, Zunt JR, Halperin JJ, Gronseth G, Roos KL. Evidence-based guideline: treatment of parenchymal neurocysticercosis: report of the Guideline Development Subcommittee of the American Academy of Neurology. Neurology. 2013;80(15):1424–9. 10.1212/WNL.0b013e31828c2f3e 23568997 PMC3662271

[13] Otte WM, Singla M, Sander JW, Singh G. Drug therapy for solitary cysticercus granuloma: a systematic review and meta-analysis. Neurology. 2013;80(2):152–62. 10.1212/WNL.0b013e31827b90a8 23269591 PMC3589189

[14] Cuello-Garcia CA, Roldan-Benitez YM, Perez-Gaxiola G, Villarreal-Careaga J. Corticosteroids for neurocysticercosis: a systematic review and meta-analysis of randomized controlled trials. Int J Infect Dis. 2013;17(8):e583–92. 10.1016/j.ijid.2012.12.010 23339852

[15] Lu G, Ades AE. Combination of direct and indirect evidence in mixed treatment comparisons. Stat Med. 2004;23(20):3105–24. 15449338 10.1002/sim.1875

[16] Caldwell DM, Ades AE, Higgins JP. Simultaneous comparison of multiple treatments: combining direct and indirect evidence. BMJ. 2005;331(7521):897–900. 16223826 10.1136/bmj.331.7521.897 PMC1255806

[17] Moher D, Liberati A, Tetzlaff J, Altman DG. Preferred reporting items for systematic reviews and meta-analyses: the PRISMA statement. PLoS Med. 2009;6(7):e1000097 10.1371/journal.pmed.1000097 19621072 PMC2707599

[18] Del Brutto OH, Rajshekhar V, White AC Jr., Tsang VC, Nash TE, Takayanagui OM, et al Proposed diagnostic criteria for neurocysticercosis. Neurology. 2001;57(2):177–83. 11480424 10.1212/wnl.57.2.177 PMC2912527

[19] Higgins J, Green S. Cochrane handbook for systematic reviews of interventions. Version 5.0.1. Cochrane Collaboration, 2008. Available: http://www.cochrane-handbook.org

[20] Dias S, Sutton AJ, Ades AE, Welton NJ. Evidence synthesis for decision making 2: a generalized linear modeling framework for pairwise and network meta-analysis of randomized controlled trials. Med Decis Making. 2013;33(5):607–17. 10.1177/0272989X12458724 23104435 PMC3704203

[21] Dias S WN, Sutton AJ, Ades AE. NICE DSU technical support document 2: a generalised linear modelling framework for pair-wise and network meta-analysis of randomised controlled trials. Available: http://www.nicedsu.org.uk. 27466657

[22] Salanti G, Ades AE, Ioannidis JP. Graphical methods and numerical summaries for presenting results from multiple-treatment meta-analysis: an overview and tutorial. J Clin Epidemiol. 2011;64(2):163–71. 10.1016/j.jclinepi.2010.03.016 20688472

[23] Cooper NJ, Sutton AJ, Morris D, Ades AE, Welton NJ. Addressing between-study heterogeneity and inconsistency in mixed treatment comparisons: Application to stroke prevention treatments in individuals with non-rheumatic atrial fibrillation. Stat Med. 2009;28(14):1861–81. 10.1002/sim.3594 19399825

[24] Chaimani A, Higgins JP, Mavridis D, Spyridonos P, Salanti G. Graphical tools for network meta-analysis in STATA. PLoS One. 2013;8(10):e76654 10.1371/journal.pone.0076654 24098547 PMC3789683

[25] Rajshekhar V, Jeyaseelan L. Seizure outcome in patients with a solitary cerebral cysticercus granuloma. Neurology. 2004;62(12):2236–40. 15210888 10.1212/01.wnl.0000130471.19171.d8

[26] Dias S WN, Sutton AJ,et al. NICE DSU Technical Support Department 3: heterogeneity, meta-regression, bias and bias adjustment. 2011. Available: http://www.nicedsu.org.uk.

[27] Baranwal AK, Singhi PD, Khandelwal N, Singhi SC. Albendazole therapy in children with focal seizures and single small enhancing computerized tomographic lesions: a randomized, placebo-controlled, double blind trial. Pediatr Infect Dis J. 1998;17(8):696–700. 9726343 10.1097/00006454-199808000-00007

[28] Chaurasia RN, Garg RK, Agarwall A, Kohli N, Verma R, Singh MK, et al Three day albendazole therapy in patients with a solitary cysticercus granuloma: a randomized double blind placebo controlled study. Southeast Asian J Trop Med Public Health. 2010;41(3):517–25. 20578537

[29] de Souza A, Thennarasu K, Yeshraj G, Kovoor JM, Nalini A. Randomized controlled trial of albendazole in new onset epilepsy and MRI confirmed solitary cerebral cysticercal lesion: effect on long-term seizure outcome. J Neurol Sci. 2009;276(1–2):108–14. 10.1016/j.jns.2008.09.010 18851861

[30] Garg RK, Potluri N, Kar AM, Singh MK, Shukla R, Agrawal A, et al Short course of prednisolone in patients with solitary cysticercus granuloma: a double blind placebo controlled study. J Infect. 2006;53(1):65–9. 16269179 10.1016/j.jinf.2005.09.002

[31] Gogia S, Talukdar B, Choudhury V, Arora BS. Neurocysticercosis in children: clinical findings and response to albendazole therapy in a randomized, double-blind, placebo-controlled trial in newly diagnosed cases. Trans Roy Soc Trop Med Hyg. 2003;97(4):416–21. 15259471 10.1016/s0035-9203(03)90075-7

[32] Kalra V, Dua T, Kumar V. Efficacy of albendazole and short-course dexamethasone treatment in children with 1 or 2 ring-enhancing lesions of neurocysticercosis: a randomized controlled trial. J Pediatr. 2003;143(1):111–4. 12915835 10.1016/S0022-3476(03)00211-7

[33] Kaur S, Singhi P, Singhi S, Khandelwal N. Combination Therapy With Albendazole and Praziquantel Versus Albendazole Alone in Children With Seizures. and Single Lesion Neurocysticercosis A Randomized, Placebo-Controlled Double Blind Trial. Pediatr Infect Dis J. 2009;28(5):403–6. 10.1097/INF.0b013e31819073aa 19325515

[34] Kishore D, Misra S. Short course of oral prednisolone on disappearance of lesion and seizure recurrence in patients of solitary cysticercal granuloma with single small enhancing CT lesion: an open label randomized prospective study. J Assoc Phys India. 2007;55:419–24. 17879495

[35] Mall RK, Agarwal A, Garg RK, Kar AM, Shukla R. Short course of prednisolone in Indian patients with solitary cysticercus granuloma and new-onset seizures. Epilepsia. 2003;44(11):1397–401. 14636346 10.1046/j.1528-1157.2003.08003.x

[36] Padma MV, Behari M, Misra NK, Ahuja GK. Albendazole in single CT ring lesions in epilepsy. Neurology. 1994;44(7):1344–6. 8035946 10.1212/wnl.44.7.1344

[37] Prakash S, Garg RK, Kar AM, Shukla R, Agarwal A, Verma R, et al Intravenous methyl prednisolone in patients with solitary cysticercus granuloma: a random evaluation. Seizure. 2006;15(5):328–32. 16621618 10.1016/j.seizure.2006.03.003

[38] Pretell EJ, Garcia HH, Custodio N, Padilla C, Alvarado M, Gilman RH, et al Short regimen of praziquantel in the treatment of single brain enhancing lesions. Clin Neurol Neurosurg. 2000;102(4):215–8. 11154807 10.1016/s0303-8467(00)00110-4

[39] Sharma SR AA, Kar AM, Shukla R, Garg RK. Evaluation of role of steroid alone and with albendazole in patients of epilepsy with single-small enhancing computerized tomography lesions. Ann Indian Acad Neurol 2007;10:39–43.

[40] Singhi P, Jain V, Khandelwal N. Corticosteroids versus albendazole for treatment of single small enhancing computed tomographic lesions in children with neurocysticercosis. J Child Neurol. 2004;19(5):323–7. 15224704 10.1177/088307380401900503

[41] Singla M, Prabhakar S, Modi M, Medhi B, Khandelwal N, Lal V. Short-course of prednisolone in solitary cysticercus granuloma: a randomized, double-blind, placebo-controlled trial. Epilepsia. 2011;52(10):1914–7. 10.1111/j.1528-1167.2011.03184.x 21777229

[42] Thussu A, Chattopadhyay A, Sawhney IM, Khandelwal N. Albendazole therapy for single small enhancing CT lesions (SSECTL) in the brain in epilepsy. J Neurol Neurosurg Psychiatry. 2008;79(3):272–5. 17928325 10.1136/jnnp.2007.128058

[43] Rajshekhar V. Rate of spontaneous resolution of a solitary cysticercus granuloma in patients with seizures. Neurology. 2001;57(12):2315–7. 11756620 10.1212/wnl.57.12.2315

[44] Rajshekhar V. Albendazole therapy for persistent, solitary cysticercus granulomas in patients with seizures. Neurology. 1993;43(6):1238–40. 8170573 10.1212/wnl.43.6.1238

[45] Chandy MJ, Rajshekhar V, Prakash S, Ghosh S, Joseph T, Abraham J, et al Cysticercosis causing single, small CT lesions in Indian patients with seizures. Lancet. 1989;1(8634):390–1. 2563549 10.1016/s0140-6736(89)91771-6

[46] Gonzalez AE, Falcon N, Gavidia C, Garcia HH, Tsang VC, Bernal T, et al Time-response curve of oxfendazole in the treatment of swine cysticercosis. Am J Trop Med Hyg. 1998;59(5):832–6. 9840607 10.4269/ajtmh.1998.59.832

[47] Robinson P, Garza A, Weinstock J, Serpa JA, Goodman JC, Eckols KT, et al Substance P causes seizures in neurocysticercosis. PLoS Pathog. 2012;8(2):e1002489 10.1371/journal.ppat.1002489 22346746 PMC3276565

[48] Takayanagui OM, Lanchote VL, Marques MP, Bonato PS. Therapy for neurocysticercosis: pharmacokinetic interaction of albendazole sulfoxide with dexamethasone. Ther Drug Monitor. 1997;19(1):51–5. 9029747 10.1097/00007691-199702000-00009

[49] Matthaiou DK, Panos G, Adamidi ES, Falagas ME. Albendazole versus praziquantel in the treatment of neurocysticercosis: a meta-analysis of comparative trials. PLoS Negl Trop Dis. 2008;2(3):e194 10.1371/journal.pntd.0000194 18335068 PMC2265431

[50] Garcia HH, Lescano AG, Lanchote VL, Pretell EJ, Gonzales I, Bustos JA, et al Pharmacokinetics of combined treatment with praziquantel and albendazole in neurocysticercosis. Br J Clin Pharmacol. 2011;72(1):77–84. 10.1111/j.1365-2125.2011.03945.x 21332573 PMC3141188

