# Supplementary material for: Albendazole and Corticosteroids for the Treatment of Solitary Cysticercus Granuloma: A Network Meta-analysis
Source: PLoS Negl Trop Dis. 2016 Feb 5;10(2):e0004418. doi: 10.1371/journal.pntd.0004418 (PMC4744042; doi:10.1371/journal.pntd.0004418)
Supplement: S1 Table — (PDF) [file pntd.0004418.s002.pdf]

S1 Table. Pooled odds ratios for seizure recurrence in sensitivity analysis after removal of the clinical trial by Singhi at el.

**Albendazole + corticosteroid**	**0.84 (0.37, 2.00)**<br>0.86 (0.42, 1.79)	**0.31 (0.11, 0.76)***<br>0.31 (0.11, 0.89)*	**0.23 (0.05, 0.92)***<br>NA
	**Corticosteroid**	**0.37 (0.17, 0.68)***<br>0.38 (0.20, 0.74)*	**0.27 (0.07, 0.93)***<br>NA
Between-study variance: 0.16		**Conservative treatment**	**0.73 (0.23, 2.23)**<br>0.76 (0.31, 1.89)
Posterior mean residual deviance: 22.28[a]			**Albendazole**

[a] Compared with 24 data points.

The column treatment is compared with the row treatment. In each cell, the first line in bold type is the odds ratio calculated by Bayesian network meta-analysis, while the second line is the odds ratio calculated by conventional pairwise meta-analysis.

Asterisks mark odds ratios with statistical significance.

NA = not applicable.
