# Supplementary figures and images for: Albendazole and Corticosteroids for the Treatment of Solitary Cysticercus Granuloma: A Network Meta-analysis
Source: PLoS Negl Trop Dis. 2016 Feb 5;10(2):e0004418. doi: 10.1371/journal.pntd.0004418 (PMC4744042; doi:10.1371/journal.pntd.0004418)

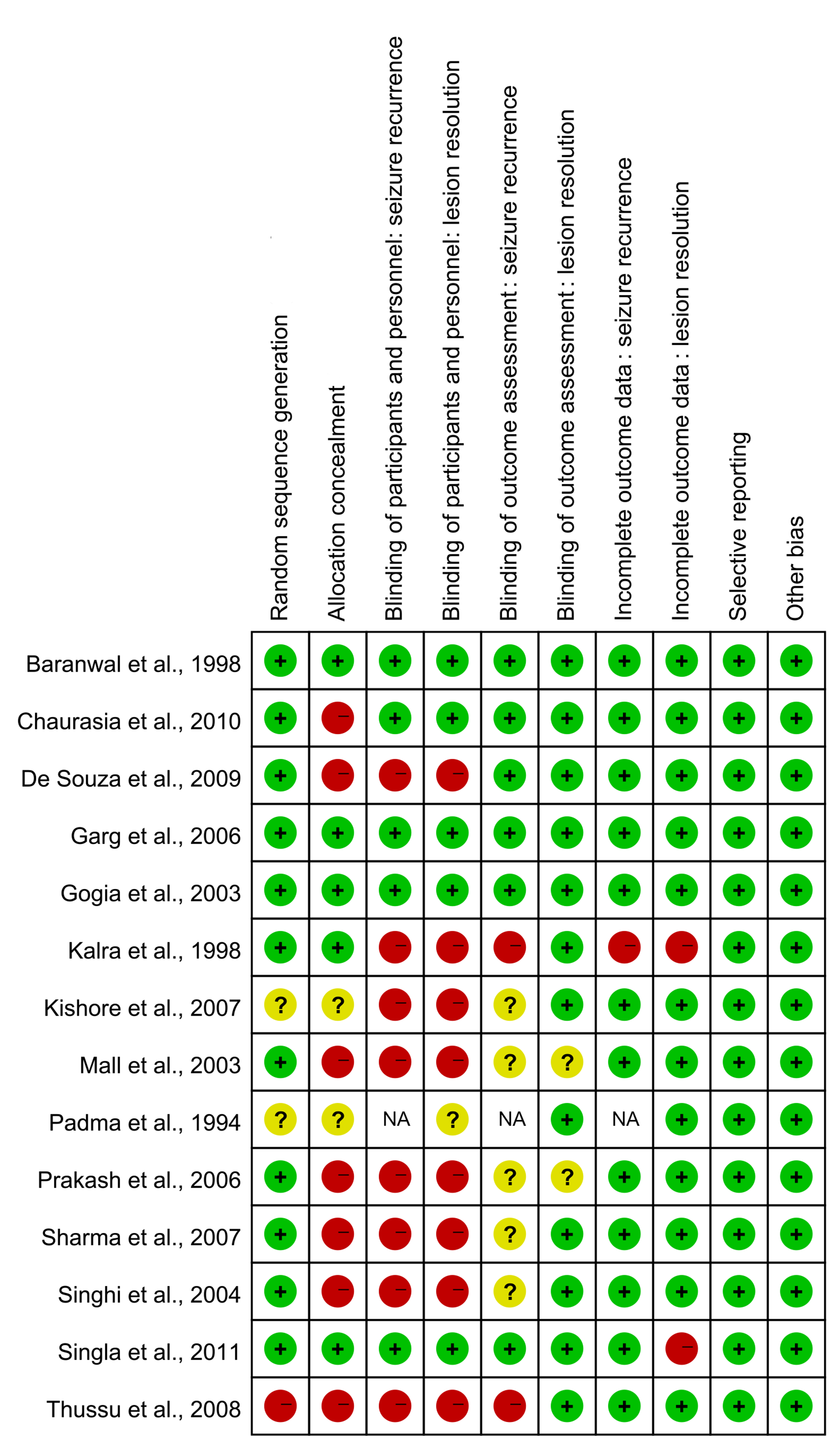

Supplement: S1 Fig — (TIF) [file pntd.0004418.s003.tif]

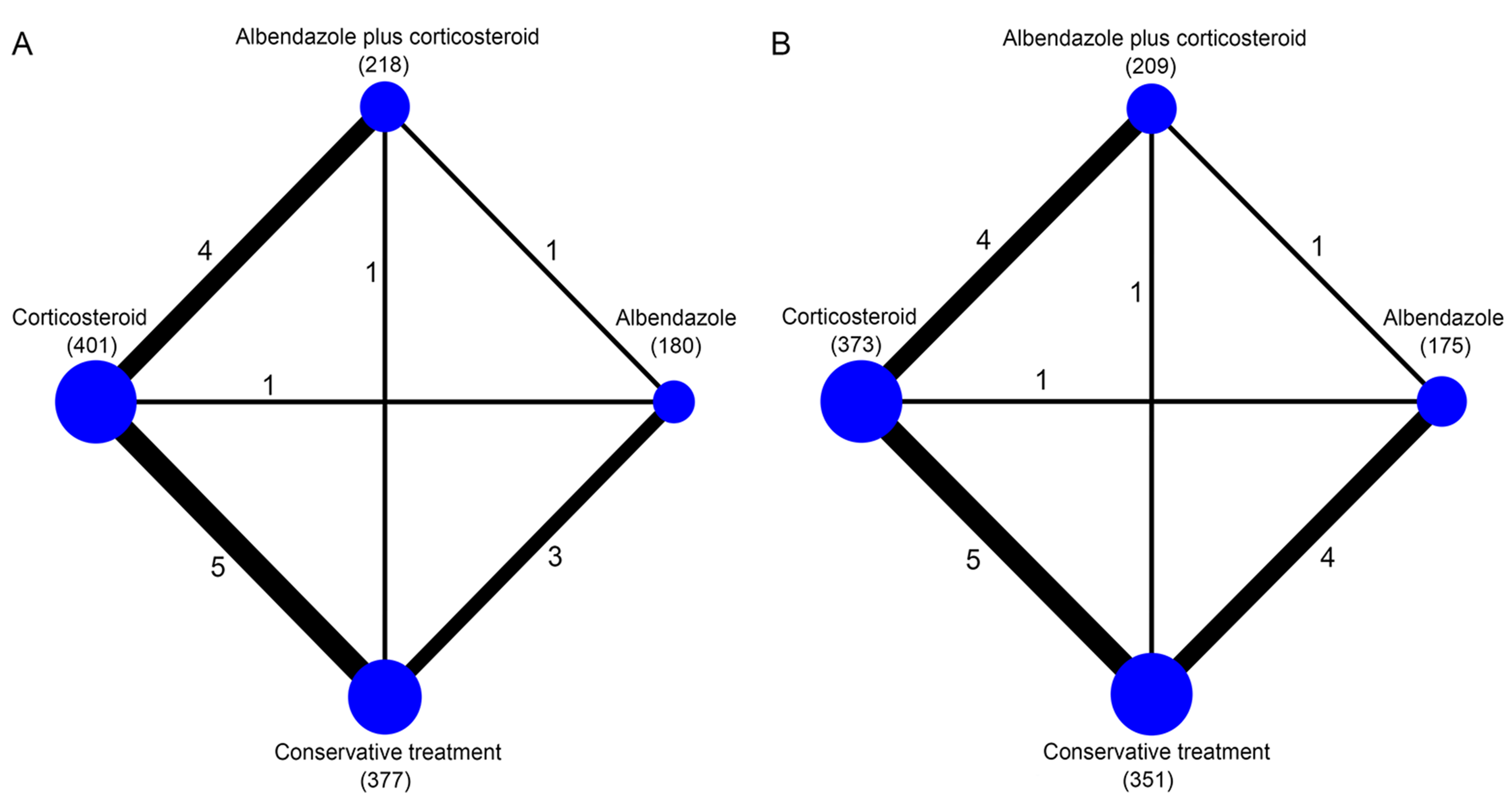

Supplement: S2 Fig — The size of the nodes is proportionate to the number of patients (in parentheses) randomized to the treatment. The width of the lines is proportionate to the number of direct comparisons (beside the lines) between the connected treatments. (TIF) [file pntd.0004418.s004.tif]

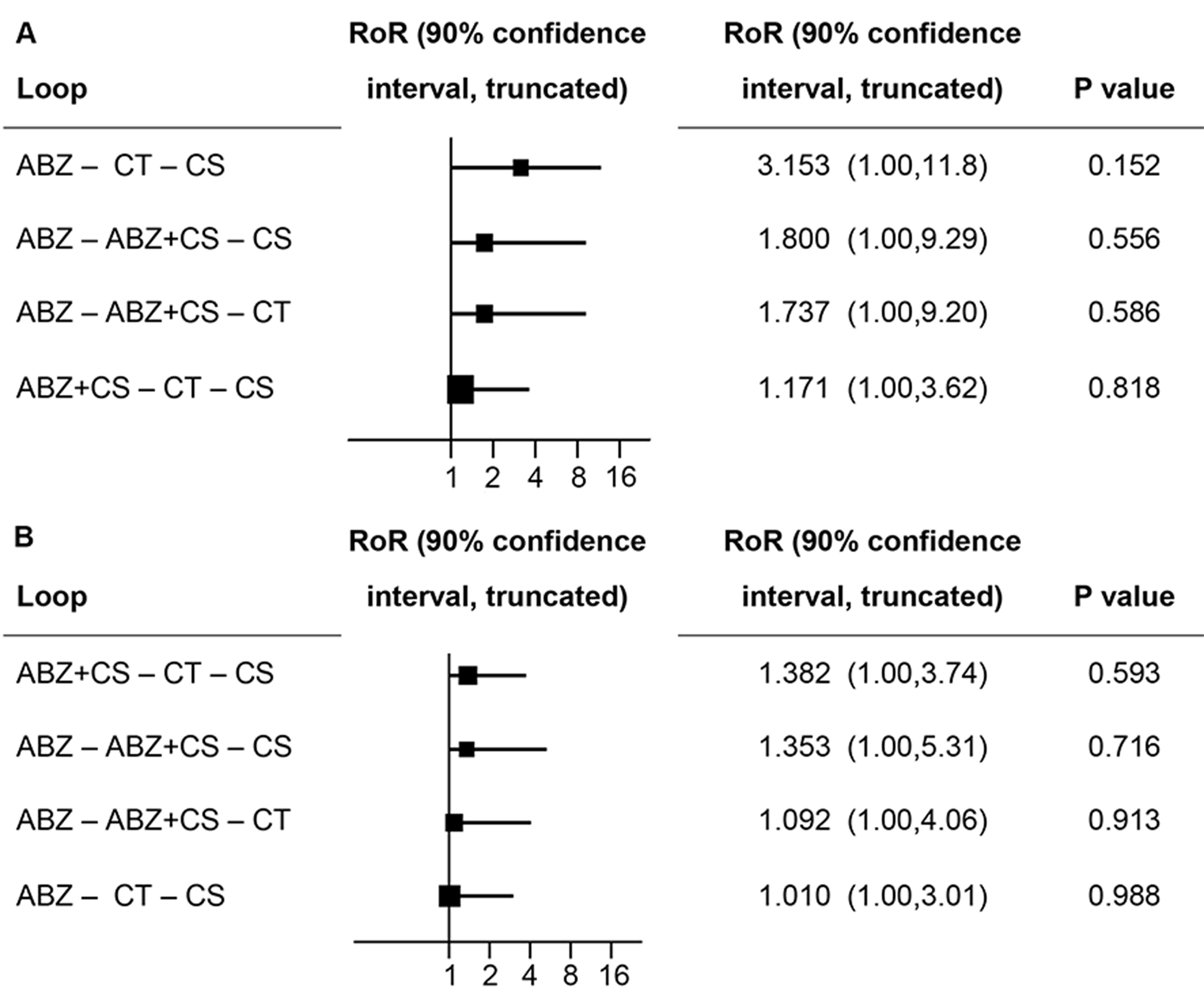

Supplement: S3 Fig — In a total of 8 loops of the two networks, none showed statistically significant inconsistency since all confidence intervals for RoRs were compatible with zero inconsistency (RoR = 1). However, for the loop “ABZ–CT–CS” in the network of seizure recurrence, the mean RoR is larger than 3, meaning that the direct estimate can be three times as large as the indirect estimate or the opposite. ABZ: albendazole, CS: corticosteroids, CT: conservative treatment, RoR: ratio of odds ratios. (TIF) [file pntd.0004418.s005.tif]

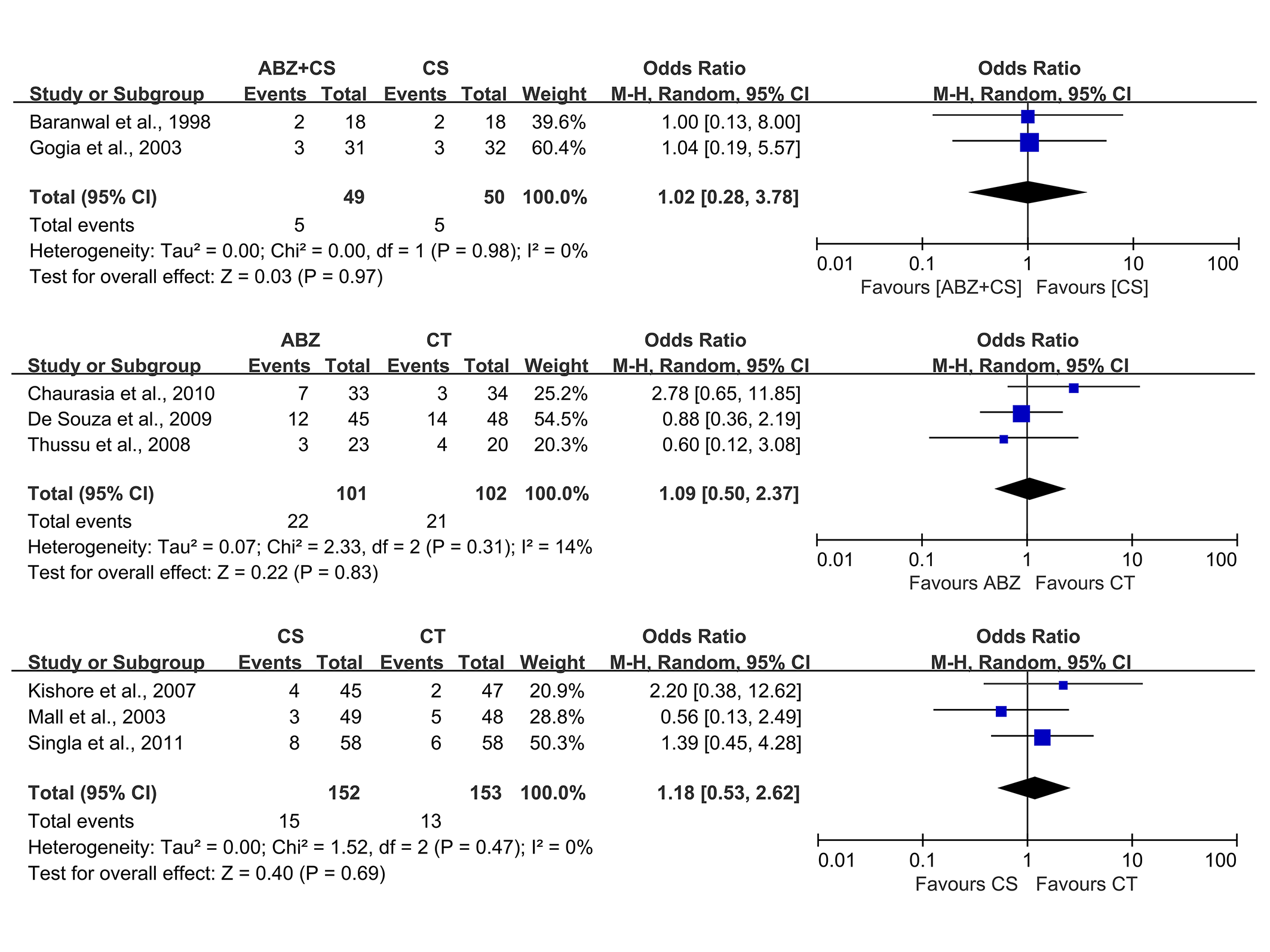

Supplement: S4 Fig — ABZ: albendazole, CS: corticosteroids, CT: conservative treatment. (TIF) [file pntd.0004418.s006.tif]
